# Impact of Air Temperature and Humidity on Performance of Heat-Source-Free Water-Floating Single-Walled Carbon Nanotube Thermoelectric Generators for IoT Sensors

**DOI:** 10.3390/s25247445

**Published:** 2025-12-07

**Authors:** Yuto Nakazawa, Tetsuya Takizawa, Takumi Nakajima, Keisuke Uchida, Masayuki Takashiri

**Affiliations:** Department of Materials Science, Tokai University, Hiratsuka 259-1292, Kanagawa, Japan; 4cajm045@tokai.ac.jp (Y.N.); 4cajm039@tokai.ac.jp (T.T.); 4cajm046@tokai.ac.jp (T.N.); 5cajm008@tokai.ac.jp (K.U.)

**Keywords:** water-floating SWCNT-TEGs, relative humidity, temperature, IoT sensor, energy harvesting

## Abstract

Thermoelectric generators (TEGs), which can generate electricity simply by floating in water, have high potential for application as power supplies of IoT sensors. However, few studies on single-wall carbon nanotube (SWCNT)-TEGs have examined the effects of the power generation environment. Therefore, we investigated the impact of the relative humidity and temperature on the TEG performance. The SWCNT-TEGs were measured in environments with air temperature controlled at 25–40 °C and relative humidity controlled at 50–90%. Evaporative cooling occurs under environmental conditions with lower relative humidity and higher temperatures, resulting in higher output voltages. The SWCNT-TEG output voltage at 50% relative humidity and 40 °C was 0.26 mV, which was approximately 1.6 times higher than that measured at the same relative humidity and 30 °C, and approximately 1.4 times higher than that measured at 80% relative humidity and the same temperature, because a lower relative humidity and higher temperature increase the amount of water vapor in the air. This facilitates evaporative cooling, increasing the temperature difference within the film, thus increasing the output voltage. These results suggest that environmental factors have a significant impact on the SWCNT-TEGs and that power generation performance can be enhanced through effective use of evaporative cooling.

## 1. Introduction

In recent years, advances in the Internet of Things (IoT) technology have led to the widespread adoption of small sensors and electronic devices [1,2,3,4,5,6,7,8]. The hardware components of IoT technology include a microprocessor unit, communication modules, and sensors. The software components include data acquisition and storage, data processing, firmware, user interfaces, and device control functions. Among the IoT technologies, sensors are the most critical factor. Long-term stable operation of the sensors requires the development of power-supply technologies that do not rely either on conventional batteries (which must be replaced) or on external power sources. One promising approach for the development of such power-supply devices is the use of energy-harvesting technologies that collect energy directly from the environment and convert it into electricity [9,10,11,12,13,14]. Among these technologies, thermoelectric generators (TEGs), which directly convert thermal energy into electricity, are particularly promising because they do not require moving parts, are maintenance-free, and can be miniaturized [15,16,17,18,19].

Inorganic materials such as bismuth telluride-based alloys have been widely used as conventional thermoelectric materials [20,21,22,23,24,25,26,27,28,29]. While these materials exhibit a relatively high thermoelectric conversion efficiency, they are quite brittle, which makes their application to flexible sheets challenging. Additionally, the use of these materials in devices faces challenges of resource limitations, high manufacturing costs, and poor environmental compatibility. Thus, it is necessary to develop environmentally friendly, flexible, lightweight, and easily processable thermoelectric materials.

Single-walled carbon nanotubes (SWCNTs) have attracted considerable research attention owing to their excellent electrical conductivity and thermoelectric properties, as well as good chemical stability and mechanical flexibility [30,31,32,33,34,35,36,37,38]. These characteristics make the SWCNTs promising materials for flexible TEGs [39,40,41,42,43,44,45,46,47,48]. Wei et al. designed an S-shaped TEG composed of SWCNTs and organic materials to harvest heat from the human body by exploiting a vertical temperature gradient [39]. Mytafides et al. fabricated a fully printed all-carbon organic TEG with outstanding flexibility and power output [40]. Norimasa et al. created a self-generated temperature gradient under uniform heating in *p*–*i*–*n* junction SWCNT-TEGs and generated electricity [41].

In our previous studies, we have developed heat-source-free water-floating SWCNT-TEGs [49,50]. Conventional TEGs require heat sources to generate temperature differences. However, water-floating SWCNT-TEGs can generate a temperature difference without a heat source by merely floating on water. When portions of the SWCNT films in the TEGs are in contact with water, the water penetrates the films by capillary action. The temperatures of these areas decrease because of evaporative cooling, thus creating a temperature difference that generates an output voltage via the Seebeck effect. As the heat flow direction can be freely controlled by changing the layout of the area where evaporative cooling occurs, TEGs can be constructed using either *p*-type or *n*-type thermoelectric material. This characteristic is beneficial for SWCNT-TEGs because of the challenging fabrication of air-stable *n*-type SWCNT films [51,52,53,54,55]. Therefore, water-floating SWCNT-TEGs, which utilize only *p*-type SWCNT films, can exploit the surrounding environment to produce a stable temperature gradient and maximize the power generation performance. Although the effect of water temperature on the performance of water-floating SWCNT-TEGs was described in our previous report [49], the effects of other environmental conditions, such as air temperature and relative humidity, have not been examined to date.

This study aimed to elucidate the effect of the aforementioned environmental conditions on the power generation performance of water-floating SWCNT-TEGs via evaporative cooling to provide design guidelines for SWCNT-TEGs suitable for various environmental conditions. The findings of this study will contribute significantly to the application and development of SWCNT-TEGs as a distributed power source technology in various IoT sensors, such as temperature, humidity, light, and water quality sensors.

## 2. Materials and Methods

**Figure 1:**
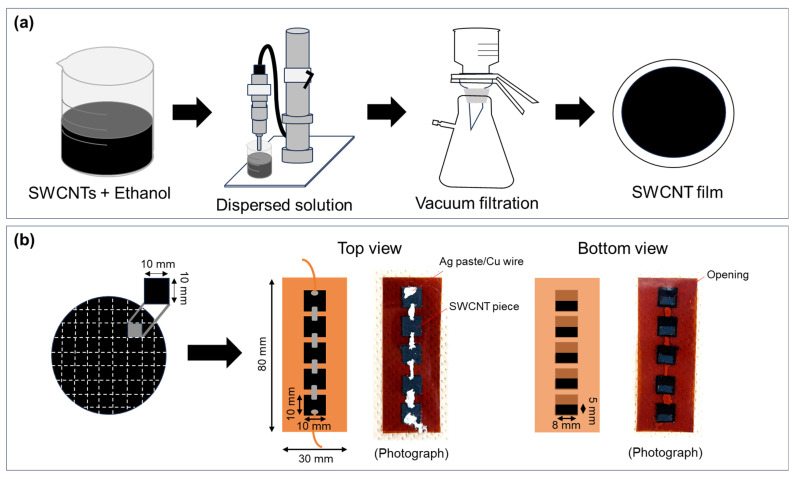
Fabrication of (**a**) SWCNT film and (**b**) SWCNT-TEG.

Figure 1a illustrates the procedure for the fabrication of the SWCNT films. SWCNT powders (0.1 g, ZEONANO SG101, ZEON, Tokyo, Japan) synthesized by the super-growth method [56] were mixed with ethanol (50 mL) (Fujifilm Wako, Tokyo, Japan) to produce the SWCNT ink, and the SWCNT ink was uniformly dispersed in an ice bath for 30 min using an ultrasonic homogenizer (Branson Sonifier SFX 250; Emerson, St. Louis, MO, USA) at an amplitude of 60% (nominal power of 200 W). The dispersion conditions were as follows: an ultrasonic amplitude of 90 mm, a frequency of 20 kHz, and a horn tip diameter of 12.7 mm. After preparing the SWCNT ink, SWCNT films were fabricated using vacuum filtering. To produce the SWCNT films, the ink was dropped in 10 mL portions onto a membrane filter (PETE, 90 mm diameter: ADVANTEC, Tokyo, Japan) placed in a suction bottle and was evenly dispersed to obtain even film thickness. The filtration was conducted under a suction pressure of 0.08 MPa for approximately 1 h. The resulting SWCNT film had a diameter of 80 mm and a thickness of approximately 70 μm. Figure 1b illustrates the fabrication process of the water-floating SWCNT-TEG. The SWCNT-TEG was fabricated using five SWCNT films measuring 10 mm × 10 mm, which were cut from the SWCNT film. An 80 mm × 30 mm polyimide sheet (Kapton, DuPont, Wilmington, DE, USA) with a thickness of 125 µm was used as the substrate. Five rectangular openings, each measuring 5 mm × 8 mm, were cut into the polyimide sheet. Subsequently, the five cut SWCNT films were attached to the openings using a two-sided polyimide tape such that only half of each film was in contact with water. Finally, the films were connected in series using thin copper wires and silver paste was attached to both ends. These wires provided external power.

The nanostructures of the SWCNT powders were examined by field-emission transmission electron microscopy (FE-TEM; JEM-2100F, JEOL, Akishima, Japan) at an accelerating voltage of 200 kV. Additionally, the surface morphologies and microstructures of the SWCNT films were examined using field-emission scanning electron microscopy (FE-SEM; S-4800, Hitachi, Tokyo, Japan). The Seebeck coefficient (*S*) of the SWCNT films in the in-plane direction was evaluated at approximately 300 K with an accuracy of ±5% [50], where one end of the film was connected to a heat sink and the other to a heater. The Seebeck coefficient was determined by calculating the ratio of the potential difference across the film to the difference between the temperatures measured using two 0.1 mm-diameter K-type thermocouples pressed against the film. Three measurements were performed for each sample, and the obtained values were averaged. The electrical conductivity (*σ*) of the SWCNT films in the in-plane direction was examined at a temperature of approximately 300 K using the four-point probe method (RT-70V, Napson, Tokyo, Japan) with an accuracy of ±3% [50]. Electrical conductivity was measured three times per sample, and the obtained values were averaged. The power factor *σS*^2^ in the in-plane direction was calculated based on the measured Seebeck coefficient and electrical conductivity values.

**Figure 2:**
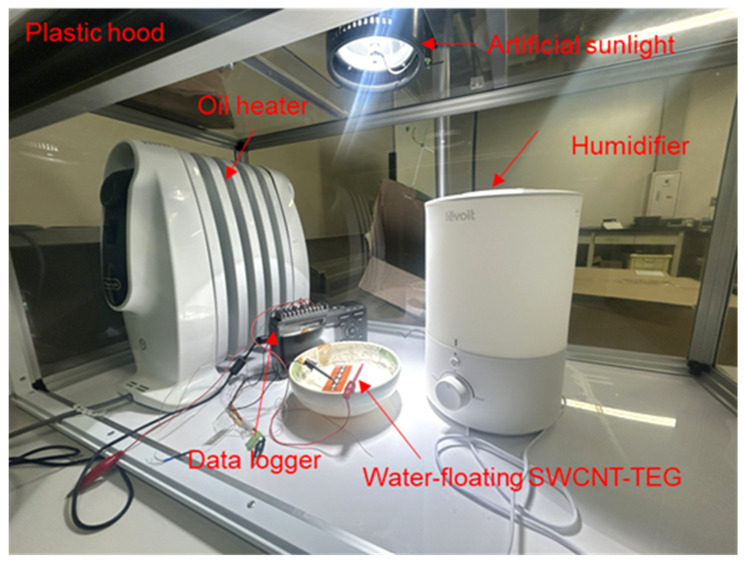
Photograph of experimental setup for water-floating SWCNT-TEG.

Figure 2 shows a photograph of the experimental setup for the water-floating SWCNT-TEG. To control the air temperature and relative humidity, the SWCNT-TEG was placed in a plastic hood (F-1000, SANPLATEC, Osaka, Japan), which contained an artificial sunlight source (SOLAX 100 W XC-100 B, SERIC, Koshigaya, Japan), an oil heater (NJ0505E, DeLonghi, Treviso, Italy), and a humidifier (Dual150 LUH-D302-WJP, Levoit, Anaheim, CA, USA). After floating the SWCNT-TEG on water (400 mL) in a plastic bowl at an approximate temperature of 43 °C, air temperature (25 °C to 40 °C) and relative humidity (50% to 90%) were independently adjusted. SWCNT-TEG was exposed to artificial sunlight at an illumination intensity of 1000 W/cm^2^, and the output voltage was measured for 30 min using a data logger (GL240, GRAPHTEC, Yokohama, Japan). The temperature distribution of the SWCNT-TEG during the artificial sunlight irradiation was measured using a thermographic camera (OPTXI40LTF20CFKT090, OPTRIS, Berlin, Germany) with a spatial resolution of 382 × 288 pixels and a temperature resolution of 0.08 °C.

## 3. Results

### 3.1. Properties of SWCNT Powders and Films

**Figure 3:**
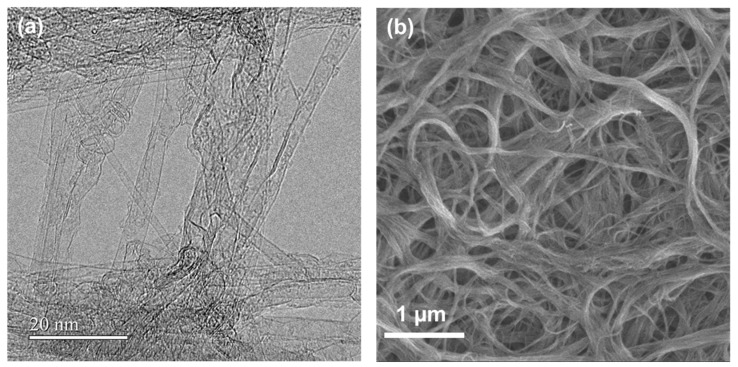
(**a**) TEM image of SWCNT powders and (**b**) SEM image of SWCNT film.

The nanostructures of the SWCNT powders analyzed using FE-TEM are shown in Figure 3a. Most of the powder consisted of SWCNTs, also containing a few mixed multi-walled carbon nanotubes. Several dozen SWCNTs were measured based on the TEM images using a ruler, and their diameters were determined to be 3–5 nm. Figure 3b shows that the orientations of the SWCNT bundles within the film are predominantly confined to the plane of the film, showing a meandering configuration, as demonstrated in the SEM image. The SWCNT bundles were more than 5 µm long, with an average diameter of approximately 200 nm. Most of the bundles had diameters ranging from 50 to 300 nm, indicating that each bundle consisted of a few hundred to a few thousand individual SWCNTs. Table 1 lists the thermoelectric properties of the SWCNT film. The SWCNT film demonstrated a *p*-type Seebeck coefficient of 54.2 µV/K, which is higher than that of the SWCNT films fabricated from SWCNT powders synthesized using methods other than the super-growth method [57,58,59,60]. This favors the high output voltage obtained for the water-floating SWCNT-TEGs. The electrical conductivity and power factor of the SWCNT films were measured to be 27.7 S/cm and 8.2 µW/(m·K^2^), respectively.

### 3.2. Performance of Water-Floating SWCNT-TEGs at Different Environmental Conditions

**Figure 4:**
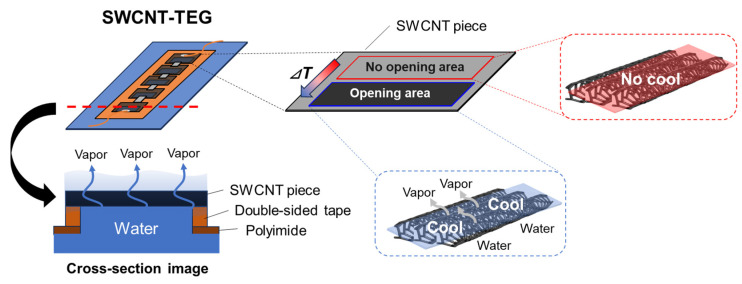
Operation mechanism of the water-floating SWCNT-TEGs.

Before explaining the performance of the water-floating SWCNT-TEG, we present its operating mechanism in Figure 4. By shielding the openings in the polyimide substrate with SWCNT films measuring approximately twice the area of the openings, areas that can and cannot absorb water were created in the SWCNT films. When the SWCNT-TEG floated on water, capillary action caused the absorbent sections of the SWCNT films to absorb water. Subsequently, the water reached the surface of the SWCNT film and evaporated. Conversely, in areas that cannot absorb water, evaporative cooling did not occur and the temperature of the areas did not decrease. During this process, a temperature difference was generated in the SWCNT films. Furthermore, the direction of the temperature difference can be controlled by changing the positions of the openings in the polyimide substrate. This enables the generation of efficient power using either *p*- or *n*-type SWCNTs.

**Figure 5:**
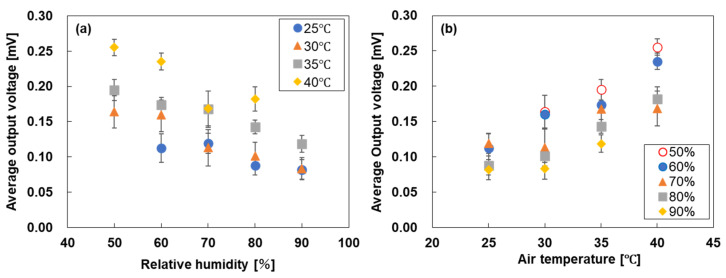
Output voltage of the water-floating SWCNT-TEGs plotted versus (**a**) relative humidity and (**b**) air temperature.

Figure 5 shows the output voltage of the water-floating SWCNT-TEG under various environmental conditions. The output voltage represents the average value evaluated over 30 min under artificial sunlight irradiation, and the error bars indicate the standard deviation. Note that the environmental conditions of an air temperature of 25 °C and 50% relative humidity, as well as the corresponding values of 40 °C and 90%, were not achieved due to the weather conditions and insufficient capacity of the humidifier. Figure 5a shows the output voltage of the SWCNT-TEG as a function of relative humidity at a constant air temperature. For the entire temperature range from 25 °C to 40 °C, the output voltage decreased as the relative humidity increased. For example, at an air temperature of 35 °C, the output voltage decreased linearly from 0.20 to 0.12 mV when the relative humidity changed from 50% to 90%, and similar trends were observed for other air temperatures. This is because the ability of the SWCNT-TEG to cool the SWCNT films via evaporative cooling was not maximized. The higher the humidity, the greater was the number of water molecules in air, which hindered evaporation from the SWCNT surface. Consequently, the temperature difference within the SWCNT films decreases, as does the output voltage. Figure 5b shows the output voltage of the SWCNT-TEG as a function of the air temperature at a constant relative humidity. For the entire relative humidity range of 50–90%, the output voltage increased with the air temperature. At a relative humidity of 60%, the output voltage increased linearly from 0.11 to 0.24 mV when the air temperature changed from 25 °C to 40 °C, and similar trends were observed for the other humidity values. Therefore, even for a constant relative humidity, the output voltage of the SWCNT-TEG changes significantly with fluctuations in the air temperature. This is because the high air temperature stimulated evaporation on the SWCNT surface, thereby promoting cooling through evaporation. This increases the temperature difference within the SWCNT films and improves the output voltage. To summarize the results presented in Figure 5a,b, the performance of the water-floating SWCNT-TEGs was strongly influenced by the relative humidity and air temperature. The highest output voltage of 0.26 mV was obtained at an air temperature of 40 °C and a relative humidity of 50%, whereas the lowest output voltage of 0.08 mV was obtained at an air temperature of 25 °C and a relative humidity of 90%. These results indicate that under conditions of low relative humidity and high temperature, the water on the film surface evaporates easily, promoting evaporative cooling. Consequently, both the temperature difference inside the film and the generated voltage increase. Conversely, under high relative humidity and low temperature conditions, the opposite phenomenon occurs. Therefore, in a natural environment where water-floating SWCNT-TEGs are expected to be used as power sources for IoT sensors, the output is likely to change by more than three times.

## 4. Discussion

**Figure 6:**
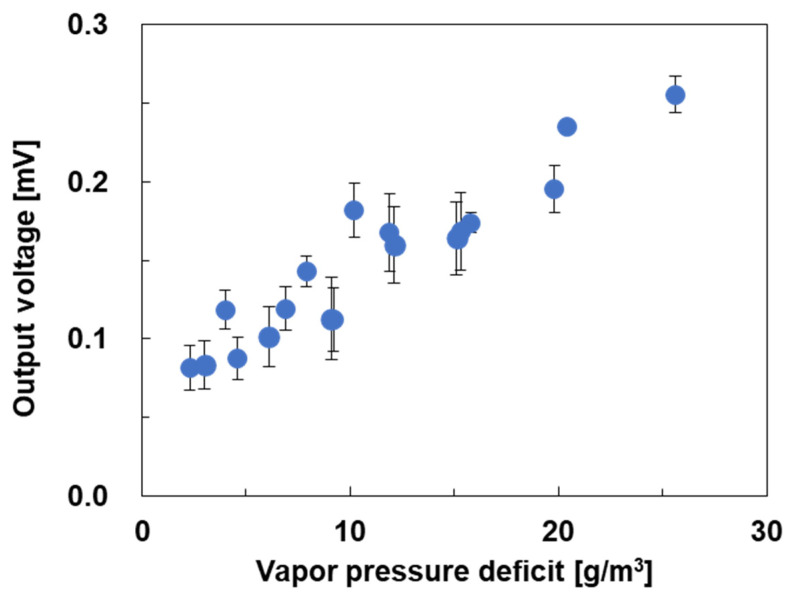
Relationship between the *VPD* and the output voltage of the water-floating SWCNT-TEGs.

To further elucidate the relationship between the performance of the water-floating SWCNT-TEGs and environmental conditions, we estimated the vapor pressure deficit (*VPD*), which is defined as the difference between the saturated amount of water vapor in the air and the actual amount of water vapor. A large *VPD* indicates that there is more water vapor in the air, making evaporation more likely. In other words, a high *VPD* promotes evaporation from the film surface and enhances evaporative cooling. *VPD* (g/m^3^) was calculated as follows [61]:

(1) $$VPD=6.1078\times exp\left(\frac{17.27T_{a}}{T_{a}+237.3}\right)\times \left(1-\frac{H_{R}}{100}\right)\times \left(\frac{216.674}{T_{a}+273.15}\right),$$

where *T_a_* (°C) is the air temperature and *H_R_* (%) is the relative humidity. The relationship between *VPD* and the output voltage of the water-floating SWCNT-TEGs is shown in Figure 6. In this study, the *VPD* was varied from 2.3 to 25.6 g/m^3^, and the output voltage increased linearly with increasing *VPD*. This is because a higher *VPD* results in greater amounts of water vapor in air, thus promoting evaporation from the SWCNT surface. This result suggests that *VPD* magnitude is the dominant factor in determining the performance of water-floating SWCNT-TEGs. Although evaporative cooling, which is affected by *VPD*, has been applied in ecology and agriculture [62,63,64,65,66,67], few energy-related applications based on *VPD* have been reported.

**Figure 7:**
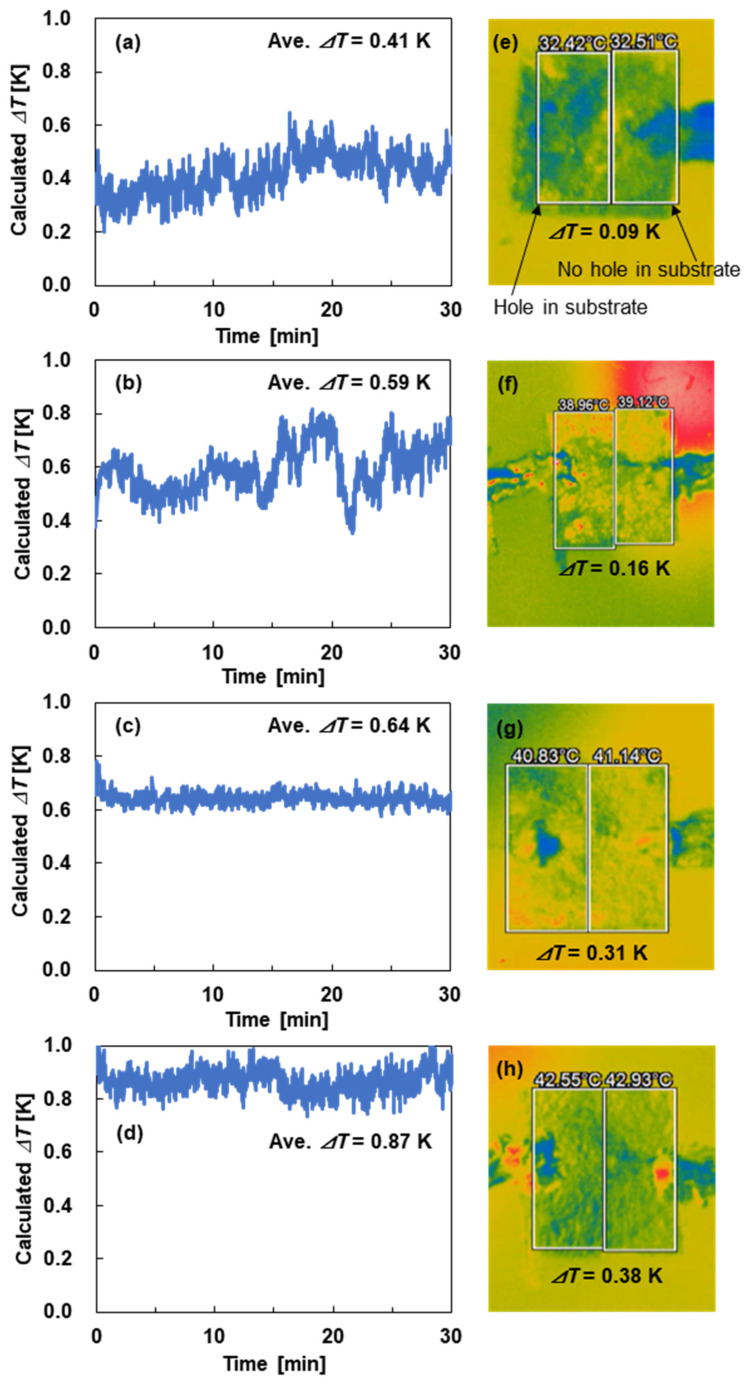
Time dependence of the calculated temperature difference in SWCNT-TEGs under artificial sunlight irradiation for a constant relative humidity of 60% at various air temperatures of (**a**) 25 °C, (**b**) 30 °C, (**c**) 35 °C, and (**d**) 40 °C. Thermographic images of the temperature distribution of SWCNT-TEGs at various air temperatures of (**e**) 25 °C, (**f**) 30 °C, (**g**) 35 °C, and (**h**) 40 °C at a constant relative humidity of 60%.

The temperature difference produced in the SWCNT film of the SWCNT-TEG was calculated based on the measured output voltage of the SWCNT-TEG and the Seebeck coefficient of the SWCNT film. In addition, the temperature distributions of the SWCNT films were analyzed using a thermographic camera. Figure 7 shows the time dependence of the calculated temperature difference under artificial sunlight irradiation and the temperature distribution of the SWCNT-TEGs at typical environmental conditions where the air temperature was varied from 25 °C to 40 °C while maintaining the relative humidity of 60%. The calculated temperature difference at an air temperature of 25 °C was 0.41 K, as shown in Figure 7a, and increased with increasing air temperature, as shown in Figure 7b,c. At an air temperature of 40 °C, the calculated temperature difference was 0.87 K (Figure 7d), which was approximately twice that at 25 °C, confirming that the temperature difference increased with increasing air temperature. Figure 7e shows the thermographic image of the water-floating SWCNT-TEGs SWCNT film at an air temperature of 25 °C and a relative humidity of 60%. The white-enclosed area on the left shows the region where the sheet below the SWCNT film contains holes and is in contact with water, whereas the white-enclosed area on the right indicates the region of the sheet without holes, suggesting that the SWCNT film was not in contact with water. The average temperatures in the white-enclosed areas on the left and right were 32.42 °C and 32.51 °C, respectively. The temperature in the white-enclosed area on the left was lower than that in the white-enclosed area on the right because of evaporative cooling, with a temperature difference of 0.09 K. This temperature difference increased with increasing air temperature, as shown in Figure 7f,g, with the temperature difference reaching 0.38 K at the air temperature of 40 °C, as shown in Figure 7d. Thus, by analyzing the calculated temperature difference and the temperature distribution of the thermographic images, we demonstrated that the temperature difference increased with increasing air temperature at a constant relative humidity. However, the magnitude of the temperature difference determined from the thermographic images was lower than that calculated from the output voltage and Seebeck coefficient. A similar phenomenon was observed in our previous study, which can be attributed to the fact that the thermographic camera displays the average temperature over a large area [68]. In addition, we present the results for the time dependence of the calculated temperature difference and the thermographic images of the SWCNT-TEGs as the relative humidity was varied from 50% to 90% while maintaining an air temperature of 30 °C in the Supplementary Material (Figure S1). These results confirmed that the temperature difference decreased with increasing relative humidity at a constant air temperature. Finally, assuming that water-floating SWCNT-TEGs will be used in various locations, we investigated the effect of water type, including river water, rainwater, sea water, and tap water, on the performance of the SWCNT-TEGs. The results are presented in the Supplementary Material (Figure S2). In summary, the output voltage of the SWCNT-TEGs depended on the type of water used. The SWCNT-TEGs exhibited the highest output voltage when river water and tap water were used. We plan to report our detailed results in the future.

## 5. Conclusions

To investigate the effect of environmental conditions on the performance of the water-floating SWCNT-TEGs, the air temperature and relative humidity were controlled. When the air temperature was varied from 25 °C to 40 °C while the relative humidity was maintained at a constant value, the output voltage and the temperature difference in the SWCNT-TEGs increased. By contrast, when the relative humidity was varied from 50% to 90% while the air temperature was maintained at a constant value, the output voltage and temperature difference in the SWCNT-TEGs decreased. The SWCNT-TEG output voltage at 50% relative humidity and 40 °C was 0.26 mV, which was approximately 1.6 times higher than that measured at the same relative humidity and 30 °C, and approximately 1.4 times higher than that measured at 80% relative humidity and the same temperature. These results indicate that the *VPD* magnitude is the dominant factor for determining the performance of water-floating SWCNT-TEGs. In addition, a temperature difference was observed in the thermographic images of the SWCNT-TEGs. These results will be useful for the design of water-floating SWCNT-TEGs for IoT sensors, including temperature, humidity, light, and water quality sensors, that can operate in various environments.

## Figures and Tables

**Table 1:** Thermoelectric properties of WCNT film.

	S [µV/K]	*σ* [S/cm]	*PF* [μW/(m·K^2^)]
SWCNT film	54.2	27.7	8.2

## Data Availability

The data presented in this study are available on request from the corresponding author.

## Supplementary Materials

The following supporting information can be downloaded at: https://www.mdpi.com/article/10.3390/s25247445/s1: Figure S1: Time dependence of the calculated temperature difference and the thermographic images of the SWCNT-TEGs as the relative humidity changes from 50% to 90% while maintaining an air temperature of 30 °C. Figure S2: Time dependence of output voltage of the SWCNT-TEGs using various types of water.

## References

[1] Dwivedi A.D., Srivastava G., Dhar S., Singh R. (2019). A decentralized privacy-preserving healthcare blockchain for IoT. Sensors.

[2] Din I.U., Guizani M., Hassan S., Kim B.-S., Khan M.K., Atiquzzaman M. (2018). The internet of things: A review of enabled technologies and future challenges. IEEE Access.

[3] Tuoi T.T.K., Toan N.V., Ono T. (2024). Thermal energy harvester using ambient temperature fluctuations for self-powered wireless IoT sensing systems: A review. Nano Energy.

[4] Gubbi J., Buyya R., Marusic S., Palaniswami M. (2013). Internet of things (IoT): A vision, architectural elements, and future directions. Future Gener. Comput. Syst..

[5] Singh E., Meyyappan M., Nalwa H.S. (2017). Flexible graphene-based wearable gas and chemical sensors. ACS Appl. Mater. Interfaces.

[6] Shi J., Liu S., Zhang L., Yang B., Shu L., Yang Y., Ren M., Wang Y., Chen J., Chen W. (2019). Smart textile-integrated microelectronic systems for wearable applications. Adv. Mater..

[7] Zhang Q., Jin T., Cai J., Xu L., He T., Wang T., Tian Y., Li L., Peng Y., Lee C. (2022). Wearable triboelectric sensors enabled gait analysis and waist motion capture for IoT-based smart healthcare applications. Adv. Sci..

[8] Lu X., Wang P., Niyato D., Kim D.I., Han Z. (2015). Wireless networks with rf energy harvesting: A contemporary survey. IEEE Commun. Surv. Tutor..

[9] Beeby S.P., Tudor M.J., White N.M. (2006). Energy harvesting vibration sources for microsystems applications. Meas. Sci. Technol..

[10] Harb A. (2011). Energy harvesting: State-of-the-art. Renew. Energy.

[11] Ku M.-L., Li W., Chen Y., Ray Liu K.J. (2016). Advances in energy harvesting communications: Past, present, and future challenges. IEEE Commun. Surv..

[12] Ryu H., Yoon H.J., Kim S.W. (2019). Hybrid Energy Harvesters: Toward sustainable energy harvesting. Adv. Mater..

[13] Qian H., Zhou Y., Cao Z., Tang T., Deng J., Huo X., Zhou H., Wang L., Wu Z. (2025). A hybrid nanogenerator based on rotational-swinging mechanism for energy harvesting and environmental monitoring in intelligent agriculture. Sensors.

[14] Fatima N., Karimov K.S., Qasuria T.A., Ibrahim M.A. (2020). A novel and stable way for energy harvesting from Bi_2_Te_3_Se alloy based semitransparent photo-thermoelectric module. J. Alloys Compd..

[15] Jaziri N., Boughamoura A., Muller J., Mezghani B., Tounsi F., Ismail M. (2020). A comprehensive review of thermoelectric generators: Technologies and common applications. Energy Rep..

[16] Hata S., Shiraishi M., Yasuda S., Juhasz G., Du Y., Shiraishi Y., Toshima N. (2022). Green route for fabrication of water-treatable thermoelectric generators. Energy Mater. Adv..

[17] Aragones R., Joan O., Ferrer C. (2025). Transforming industrial maintenance with thermoelectric energy harvesting and NB-IoT: A case study in oil refinery applications. Sensors.

[18] Toan N.V., Tuoi T.T.K., Ono T. (2020). Thermoelectric generators for heat harvesting: From material synthesis to device fabrication. Sensors.

[19] Amma Y., Miura K., Nagata S., Nishi T., Miyake S., Miyazaki K., Takashiri M. (2022). Ultra-long air-stability of *n*-type carbon nanotube films with low thermal conductivity and all-carbon thermoelectric generators. Sci. Rep..

[20] Goldsmid H.J. (2014). Bismuth telluride and Its alloys as materials for thermoelectric generation. Materials.

[21] Harman T.C., Paris B., Miller S.E., Goering H.L. (1957). Preparation and some physical properties of Bi_2_Te_3_, Sb_2_Te_3_, and As_2_Te_3_. J. Phys. Chem. Solids.

[22] Satterthwaite C.B., Ure R.W. (1957). Electrical and thermal properties of Bi_2_Te_3_. Phys. Rev..

[23] Goldsmid H.J. (1958). The electrical conductivity and thermoelectric power of bismuth telluride. Proc. Phys. Soc..

[24] Chen Z.-G., Han G., Li L., Zou J. (2014). Point defect engineering of high-performance bismuth-telluride-based thermoelectric materials. ACS Appl. Mater. Interfaces.

[25] Hu L.-P., Zhu T.-J., Wang Y.-G., Xie H.-H., Xu Z.-J., Zhao X.-B. (2014). Shifting up the optimum figure of merit of *p*-type bismuth telluride-based thermoelectric materials for power generation by suppressing intrinsic conduction. NPG Asia Mater..

[26] Xie W., Tang X., Yan Y., Zhang Q., Tritt T.M. (2009). High thermoelectric performance alloy with unique low-dimensional structure. J. Appl. Phys..

[27] Hu L.P., Liu X.H., Xie H.H., Shen J.J., Zhu T.J., Zhao X.B. (2012). Improving thermoelectric properties of *n*-type bismuth-telluride-based alloys by deformation-induced lattice defects and texture enhancement. Acta Mater..

[28] Norimasa O., Chiba T., Hase M., Komori T., Takashiri M. (2022). Improvement of thermoelectric properties of flexible Bi2Te3 thin films in bent states during sputtering deposition and post-thermal annealing. J. Alloys Compd..

[29] Chiba T., Yabuki H., Takashiri M. (2023). High thermoelectric performance of flexible nanocomposite films based on nanoplates and carbon nanotubes selected using ultracentrifugation. Sci. Rep..

[30] Iijima S., Ichihashi T. (1993). Single-shell carbon nanotubes of 1-nm diameter. Nature.

[31] Blackburn J.L., Ferguson A.J., Cho C., Grunlan J.C. (2018). Carbon-nanotube-based thermoelectric materials and devices. Sensors.

[32] Zhang M., Cao X., Wen M., Chen C., Wen Q., Fu Q., Deng H. (2023). Highly electrical conductive flexible thermoelectric films fabricated by a high-velocity non-solvent turbulent secondary doping approach. ACS Appl. Mater. Interfaces.

[33] Khongthong J., Raginov N.I., Khabushev E.M., Goldt A.E., Kondrashov V.A., Russakov D.M., Shandakov S.D., Krasnikov D.V., Nasibulin A.G. (2024). Aerosol doping of SWCNT films with *p*- and *n*-type dopants for optimizing thermoelectric performance. Carbon.

[34] Yu M.F., Files B.S., Arepalli S., Ruoff R.S. (2000). Tensile loading of ropes of single wall carbon nanotubes and their mechanical properties. Phys. Rev. Lett..

[35] Kaempgen M., Chan C.K., Ma J., Cui Y., Gruner G. (2009). Printable thin film supercapacitors using single-walled carbon nanotubes. Nano Lett..

[36] Gojny F.H., Wichmann M.H.G., Fiedler B., Kinloch I.A., Bauhofer W., Windle A.H., Schulte K. (2006). Evaluation and identification of electrical and thermal conduction mechanisms in carbon nanotube/epoxy composites. Polymer.

[37] Seki Y., Takashiri M. (2020). Freestanding bilayers of drop-cast single-walled carbon nanotubes and electropolymerized poly(3,4-ethylenedioxythiophene) for thermoelectric energy harvesting. Org. Electron..

[38] Yonezawa S., Chiba T., Seki Y., Takashiri M. (2021). Origin of *n* type properties in single wall carbon nanotube films with anionic surfactants investigated by experimental and theoretical analyses. Sci. Rep..

[39] Wei S., Liu L., Huang X., Zhang Y., Liu F., Deng L., Bilotti E., Chen G. (2022). Flexible and foldable films of SWCNT thermoelectric composites and an s-shape thermoelectric generator with a vertical temperature gradient. ACS Appl. Mater. Interfaces.

[40] Mytafides C.K., Tzounis L., Karalis G., Formanek P., Paipetis A.S. (2021). High-power all-carbon fully printed and wearable SWCNT-based organic thermoelectric generator. ACS Appl. Mater. Interfaces.

[41] Norimasa O., Tamai R., Nakayama H., Shinozaki Y., Takashiri M. (2025). Self-generated temperature gradient under uniform heating in *p*–*i*–*n* junction carbon nanotube thermoelectric generators. Sci. Rep..

[42] Wu R., Yuan H., Liu C., Lan J.-L., Yang X., Lin Y.-H. (2018). Flexible PANI/SWCNT thermoelectric films with ultrahigh electrical conductivity. RSC Adv..

[43] Yun J.-S., Choi S., Im S.H. (2021). Advances in carbon-based thermoelectric materials for high-performance, flexible thermoelectric device. Carbon Energy.

[44] Macleod B.A., Staton N.J., Gould I.E., Wesenberg D., Ihly R., Owczarczyk Z.R., Hurst K.E., Fewox C.S., Folmar C.N., Hughes K.H. (2017). Large *n*- and *p*-type thermoelectric power factors from doped semiconducting single-walled carbon nanotube thin films. Energy Environ. Sci..

[45] Wu G., Gao C., Chen G., Wang X., Wang H. (2016). High-performance organic thermoelectric modules based on flexible films of a novel *n*-type single-walled carbon nanotube. J. Mater. Chem. A.

[46] Meng Q., Cai K., Du Y., Chen L. (2019). Preparation and thermoelectric properties of SWCNT/PEDOT:PSS coated tellurium nanorod composite films. J. Alloys Compd..

[47] Xu C., Yang S., Li P., Wang H., Li H., Liu Z. (2022). Wet-spun PEDOT:PSS/CNT composite fibers for wearable thermoelectric energy harvesting. Compos. Commun..

[48] Li H., Liang Y., Liu S., Qiao F., Li P., He C. (2019). Modulating carrier transport for the enhanced thermoelectric performance of carbon nanotubes/polyaniline composites. Org. Electron..

[49] Chiba T., Amma Y., Takashiri M. (2021). Heat source free water floating carbon nanotube thermoelectric generators. Sci. Rep..

[50] Nakazawa Y., Yamamoto H., Okano Y., Amezawa T., Kuwahata H., Miyake S., Takashiri M. (2025). Boosting performance of heat-source free water-floating thermoelectric generators by controlling wettability by mixing single-walled carbon nanotubes with α-cellulose. Appl. Therm. Eng..

[51] Nonoguchi Y., Nakano M., Murayama T., Hagino H., Hama S., Miyazaki K., Matsubara R., Nakamura M., Kawai T. (2016). Simple salt-coordinated *n*-type nanocarbon materials stable in air. Adv. Funct. Mater..

[52] Horike S., Wei Q., Akaike K., Kirihara K., Mukaida M., Koshiba Y., Ishida K. (2022). Bicyclic-ring base doping induces *n*-type conduction in carbon nanotubes with outstanding thermal stability in air. Nat. Commun..

[53] Nakashima Y., Yamaguchi R., Toshimitsu F., Matsumoto M., Borah A., Staykov A., Islam M.S., Hayami S., Fujigaya T. (2019). Air-stable *n*-type single-walled carbon nanotubes doped with benzimidazole derivatives for thermoelectric conversion and their air-stable mechanism. ACS Appl. Nano Mater..

[54] Suzuki D., Terasaki N., Nonoguchi Y. (2025). Investigation into the stability of chemical doping and the structure of carbon nanotube. AIP Adv..

[55] Yamamoto H., Amezawa T., Okano Y., Hoshino K., Ochiai S., Sunaga K., Miyake S., Takashiri M. (2025). High thermal durability and thermoelectric performance with ultra-low thermal conductivity in *n*-type single-walled carbon nanotube films by controlling dopant concentration with cationic surfactant. Appl. Phys. Lett..

[56] Hata K., Futaba D.N., Mizuno K., Namai T., Yumura M., Iijima S. (2004). Water-assisted highly efficient synthesis of impurity-free single-walled carbon nanotubes. Science.

[57] Chung S.H., Kim D.H., Kim H., Kim H., Jeong S.W. (2020). Thermoelectric properties of PEDOT: PSS and acid-treated SWCNT composite films. Mater. Today Commun..

[58] Ji D., Li B., Raj B.T., Li X., Zhang D., Rezeq M., Cantwell W., Zheng L. (2025). In Situ Surface polymerization of PANI/SWCNT bilayer film: Effective composite for improving Seebeck coefficient and power factor. Adv. Mater. Interfaces.

[59] Liu S., Li H., He C. (2019). Simultaneous enhancement of electrical conductivity and Seebeck coefficient in organic thermoelectric SWNT/PEDOT:PSS nanocomposites. Carbon.

[60] Zheng D., Zhang J., Sun S., Liang J., Li Y., Luo J., Liu D. (2024). Flexible organic thermoelectric composites and devices with enhanced performances through fine-tuning of molecular energy levels. ACS Appl. Electron. Mater..

[61] Han X., Liu T., Cai Y., Wang D., Wei X., Hai Y., Shi R., Guo W. (2025). Design and evaluation of an innovative thermoelectric-based dehumidifier for greenhouses. Agronomy.

[62] Pelletier V., Pepin S., Gallichand J., Caron J. (2016). Reducing cranberry heat stress and midday depression with evaporative cooling. Sci. Hortic..

[63] Hao X., Sun F., Zhang J., Ci M., Li Y., Fan X., Liang Q., Li X. (2025). Vapor pressure deficit governs oasis cooling efficiency and drought intensified water-heat tradeoffs in arid regions. J. Hydrol..

[64] Zhang D., Jiao X., Du Q., Song X., Li J. (2018). Reducing the excessive evaporative demand improved photosynthesis capacity at low costs of irrigation via regulating water driving force and moderating plant water stress of two tomato cultivars. Agric. Water Manag..

[65] Pahuja R., Verma H.K., Uddin M. (2015). Implementation of greenhouse climate control simulator based on dynamic model and vapor pressure deficit controller. Eng. Agric. Environ. Food.

[66] Grossiord C., Buckley N.T., Cernusak L.A., Novick K.A., Poulter B., Siegwolf R.T.W., Sperry J.S., McDowell N.G. (2020). Plant responses to rising vapor pressure deficit. New Phytol..

[67] López J., Way A.D., Sadok W. (2021). Systemic effects of rising atmospheric vapor pressure deficit on plant physiology and productivity. Glob. Change Biol..

[68] Uchida K., Shinozaki Y., Nakayama H., Ochiai S., Nakazawa Y., Takashiri M. (2025). SWCNT/PEDOT:PSS/SA composite yarns with high mechanical strength and flexibility via wet spinning for thermoelectric applications. Sensors.

