# Supplementary material for: Impact of Air Temperature and Humidity on Performance of Heat-Source-Free Water-Floating Single-Walled Carbon Nanotube Thermoelectric Generators for IoT Sensors
Source: Sensors (Basel). 2025 Dec 7;25(24):7445. doi: 10.3390/s25247445 (PMC12736923; doi:10.3390/s25247445)
Supplement: Supplementary file 1 [file sensors-25-07445-s001.zip › sensors-3979937-supplementary.pdf]

Impact of Air Temperature and Humidity on Performance of Heat-Source-Free Water-Floating Single-Walled Carbon Nanotube Thermoelectric Generators for IoT Sensors

Yuto Nakazawa, Tetsuya Takizawa, Takumi Nakajima, Keisuke Uchida and Masayuki Takashiri *

Department of Materials Science, Tokai University, Hiratsuka 259-1292, Kanagawa, Japan; 4cajm045@tokai.ac.jp (Y.N.); 4cajm039@tokai.ac.jp (T.T.); 4cajm046@tokai.ac.jp (T.N.); 5cajm008@tokai.ac.jp (K.U.)
* Correspondence: takashiri@tokai.ac.jp

Supplementary Materials

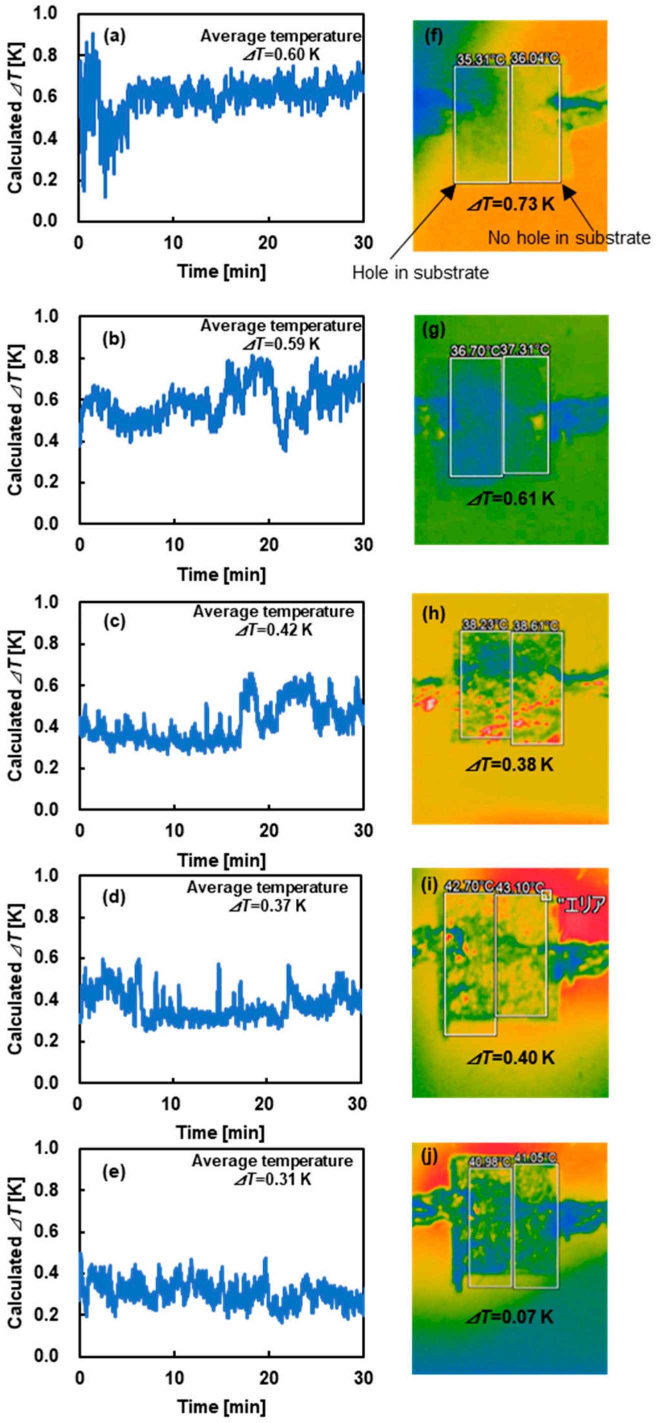

Figure S1. Time dependence of the calculated temperature difference in SWCNT-TEGs under artificial sunlight irradiation for a constant air temperature of 30°C at relative humidity of (a) 50%, (b) 60%, (c) 70%, (d) 80%, and (e) 90%. Thermographic images of the temperature distribution of SWCNT-TEGs at various relative humidities of (f) 50%, (g) 60%, (h) 70%, (i) 80%, and (j) 90% at a constant air temperature of 30°C.

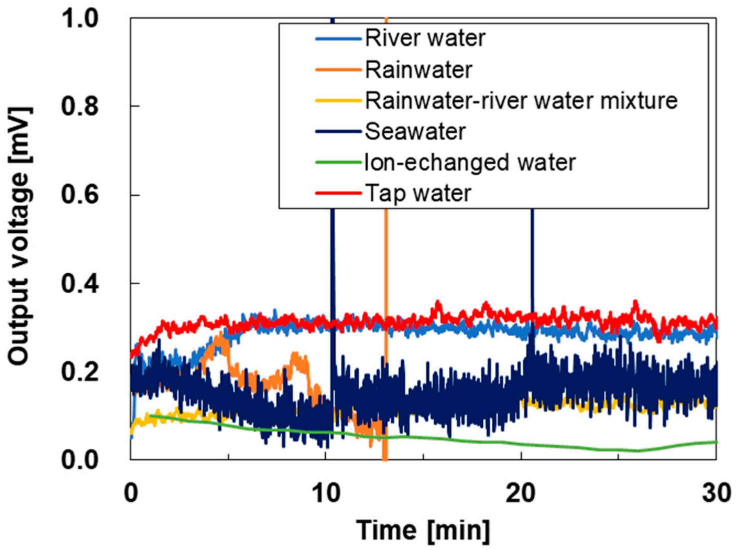

Figure S2. Time dependence of output voltage of the SWCNT-TEGs using various types of water.

In this experiment, air temperature and relative humidity were not controlled. After floating the SWCNT-TEG on various types of water (400 mL) in a plastic bowl at approximately 30 °C, including river water, rainwater, a rainwater-river water mixture, seawater, ion-exchanged water, and tap water, the SWCNT-TEG was exposed to artificial sunlight at an illumination intensity of 1000 W/cm^2. The output voltage was measured for 30 min using a data logger (GL240, GRAPHTEC, Yokohama, Japan). The SWCNT-TEGs exhibited the highest output voltage when using river water and tap water.
